# Factors associated with sedentary behavior among breast cancer patients during chemotherapy: A cross-sectional study based on COM-B model

**DOI:** 10.1097/MD.0000000000043948

**Published:** 2025-08-15

**Authors:** Ye Yang, Wei Jia, Kaiping Wu, Lingyun Xu, Yuwen Jiao

**Affiliations:** a Department of Breast Surgery, The Second People’s Hospital of Changzhou, the Third Affiliated Hospital of Nanjing Medical University, Changzhou, Jiangsu Province, China; b Changzhou Medical Center, Nanjing Medical University, Changzhou, Jiangsu Province, China; c Department of Gastrointestinal Surgery, The Second People’s Hospital of Changzhou, the Third Affiliated Hospital of Nanjing Medical University, Changzhou, Jiangsu Province, China.

**Keywords:** breast cancer, chemotherapy, COM-B model, sedentary behavior

## Abstract

Sedentary behavior (SB) is one of the prevalent unhealthy behaviors among breast cancer (BC) patients during chemotherapy and is associated with a variety of adverse health outcomes. Employing a comprehensive theoretical framework to identify the key determinants of SB is a critical step in developing targeted, evidence-based interventions. The purpose of this study was to explore the determinants of SB by using the Capability, Opportunity, Motivation – Behavior model. A cross-sectional survey was conducted and 234 BC patients during chemotherapy were conveniently recruited from a tertiary hospital. The participants completed measures of SB, shoulder function, common cancer-related symptoms, fatigue, exercise self-efficacy, depression, social support, neighborhood built environment, and sociodemographic and clinical characteristics. Descriptive statistics, univariate analysis, Pearson correlation analysis, and multiple linear regression were applied for data analysis. Multiple linear regression results showed that exercise self-efficacy, depression, fatigue, shoulder function, instrumental support, informational support, age, residence status, work status, body mass index, and cancer stage were the main influencing determinants of SB and explained 33.46% of the variance. Findings support the Capability, Opportunity, Motivation – Behavior model’s explanatory potential in the context of BC patients’ SB and also provide reasonable targets for a behavioral intervention for reducing SB.

## 1. Introduction

Breast cancer (BC) is the most commonly diagnosed type of cancer among women worldwide. In 2020, approximately 2.26 million new cases of BC were reported.^[1]^ The vast majority (86%) of BC patients receive chemotherapy after diagnosis.^[2]^ Chemotherapy is one of the most common and effective treatment options and is suitable for the treatment of multiple stages of BC. As cytotoxic agents, chemotherapy drugs can effectively achieve the purpose of systemic antitumor treatment. However, they inevitably affect normal cells, causing a series of side effects, such as nausea, vomiting, taste disorders, fatigue, secondary cardiomyopathy, and so on.^[3]^ These burdensome side effects not only adversely impact patients’ quality of life and mental state, but also frequently lead to changes in diet, exercise, and other behavioral habits. Therefore, it is important that BC patients undergoing chemotherapy avoid and reduce unhealthy behaviors that may further aggravate the side effects of chemotherapy and affect patient prognosis.

Sedentary behavior (SB) is one of the common and frequently overlooked unhealthy behaviors. SB is defined as any behavior with energy expenditure ≤1.5 metabolic equivalents in the waking state, involving all sedentary activities such as using electronic devices, writing, reading, talking, and taking a bus while sitting, leaning, or lying flat.^[4]^ It is estimated that the average daily sedentary time of BC patients during chemotherapy is more than 10 hours, and SB on the day of/posttreatment days increased over time.^[2]^ However, SB is associated with a variety of adverse health outcomes in BC patients, including aggravated depression, increased fatigue, reduced quality of life, and cancer-specific mortality.^[2]^ Considering these negative effects, it is very important to better understand the determinants of SB and to develop targeted measures for behavioral intervention.

There have been some comments on the influencing factors of SB in BC patients. These confirmed factors involve multiple levels, including demographic characteristics (age, education level), disease-related factors (fatigue, obesity), and psychological factors (negative emotions).^[5,6]^ However, the majority of studies have not been informed by a theoretical framework, which limits the comprehensive explanation of the determinants of SB. SB is a multidimensional, multicausal, and complex phenomenon. Research focusing on single causal pathways often fails to comprehensively explain the formation and change of SB, particularly among BC patients during chemotherapy who face special physical, psychological, and social challenges.

**Figure 1.**
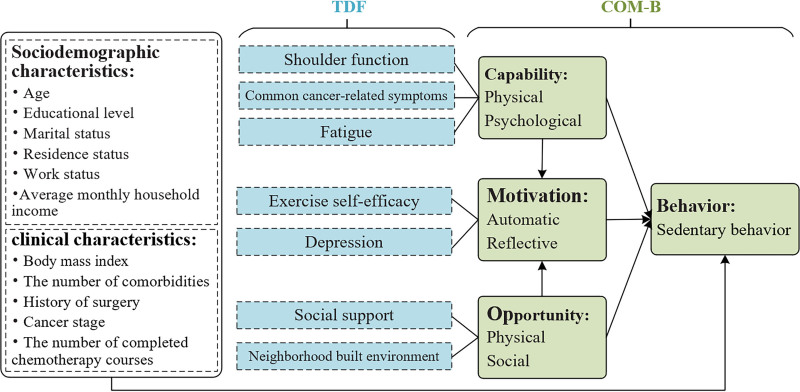
The theoretical framework guiding the study.

The Capability, Opportunity, Motivation – Behavior (COM-B) model is widely recognized as one of the most comprehensive and systematic theoretical frameworks for understanding health-related behavior.^[7]^ In previous studies, the model has been effectively employed to analyze physical inactivity in certain contexts.^[8]^ The COM-B model asserts that behavior is established through interactions between 3 main constructs: capability (the psychological or physical capability of an individual to perform a behavior), opportunity (the physical or social factors that promote or encourage behavior), and motivation (the reflective or automatic processes that guide and motivate behavior, such as conscious planning and evaluation or emotional responses).^[9]^ These 3 main constructs can directly affect behavior, while capability and opportunity can also indirectly affect behavior through motivation. In order to enhance the applicability of the COM-B model in empirical research, Michie and coworkers further subdivided the 3 main constructs into 14 domains by developing the Theoretical Domain Framework (TDF).^[10]^ The TDF is also regarded as a secondary dimension of the COM-B model because it refined the proximal determinants of behavior. In this study, based on the mapping relationship between the COM-B model and the TDF, along with insights from previous relevant literature, we classified the potential influencing factors of SB in BC patients during chemotherapy into 3 main categories: “capability, opportunity, and motivation.” Figure 1 illustrates the theoretical framework that guided our study. The objective of this study was to comprehensively examine the determinants influencing SB in BC patients during chemotherapy through the above structured approach.

## 2. Materials and methods

### 2.1. Study design

A cross-sectional study design was used.

### 2.2. Participants and sample size

From April to November 2024, participants were recruited by convenience sampling from the breast surgery department of a tertiary hospital in Jiangsu Province, China. The inclusion criteria were defined as follows: adult women (≥18 years of age) with a BC clinical diagnosis; patients who completed at least one chemotherapy cycle and were undergoing further chemotherapy; patients who agreed to participate and submitted written informed consent. Exclusion criteria were as follows: patients with mental illness, cognitive impairment, or other diseases that could interfere with their study participation; patients with distant metastasis of tumors or poor physical condition; patients with significantly impaired walking function who required long-term bed rest or use of walking assistive devices.

According to Kendall multivariable analysis sample size estimation method, the sample size should be at least 10 to 20 times the number of variables.^[11]^ In this study, the number of observed variables was 18, and the calculated sample size was 180 to 360 cases. Considering a potential 10% sample loss, the survey sample size was adjusted to at least 200 to 400 cases. Finally, 234 BC patients during chemotherapy completed the survey successfully.

### 2.3. Data collection

Researchers strictly evaluated the recruited BC patients based on predefined inclusion and exclusion criteria. They subsequently explained the objectives and significance of the study to the participants and obtained their written informed consent. The survey was conducted using a paper-based questionnaire and lasted about 25 to 30 minutes. During the investigation, researchers used unified instructions to explain the contents and filling methods of the questionnaire to the participants. Participants were then required to complete the questionnaire independently. For participants without literacy skills, researchers offered verbal assistance by providing standardized explanations while ensuring the integrity of the data collection. All questionnaires were collected in situ right after their completion. Within 24 hours after collection, researchers checked the completeness of questionnaire responses and verified the accuracy of the information by referring to medical records. Any omitted or wrongly filled items were promptly clarified with the participants and corrected as necessary.

### 2.4. Measurement

#### 2.4.1. Sociodemographic and clinical characteristics

The general information questionnaire, developed through a comprehensive process, was used to collect sociodemographic and clinical characteristics. Sociodemographic variables include age, education level, marital status, residence status, work status, and average monthly household income. In addition, the researchers also collected clinical information from the participants’ medical records. These clinical details include body mass index (BMI), number of comorbidities, history of surgery, cancer stage, and number of completed chemotherapy courses.

#### 2.4.2. Sedentary behavior

The Chinese version of the International Physical Activity Questionnaire (long self-administered format) (IPAQ) was used to assess self-reported SB in participants. This questionnaire encompasses 5 activity domains: work-related activities, transportation activities, household activities, leisure-time activities, and SB. Previous studies have evaluated the reliability and validity of the IPAQ in the Chinese community female population.^[12]^ Among them, the test-retest coefficient of sedentary dimension and the correlation coefficient of validity scale were 0.918 and 0.630, respectively.^[12]^ This study used the SB section of the IPAQ to assess the total time spent being sedentary on weekdays and non-weekdays over the past week.

#### 2.4.3. Shoulder function

The Constant–Murley Score was used to evaluate the shoulder function of the affected limb of the participants. This tool contains the following 4 subscales: pain, activities of daily living, active range of motion, and strength. Among them, the pain and activities of daily living subscales are self-reported by the participants, while the remaining subscales involve objective evaluations conducted by the researchers. The overall score ranges from 0 to 100 points, a higher score indicates better shoulder function. A previous study indicated that the Chinese version of the Constant–Murley Score had sufficient reliability, with Cronbach α of 0.739 and the intraclass correlation coefficient of 0.827.^[13]^

#### 2.4.4. Common cancer-related symptoms

The Chinese version of the M. D. Anderson Symptom Inventory was used to assess the common cancer-related symptoms. The scale is divided into 2 parts. The first part assesses the severity of common symptoms in cancer patients, and the second part evaluates interference with their daily lives. In this study, only the first part was used to assess the severity of 13 common core symptoms in participants within the past 7 days. Each item is scored on a numerical scale from 0 to 10, with higher scores indicating more severe symptoms. The Chinese version of the M. D. Anderson Symptom Inventory has shown good reliability and validity in BC patients. The Cronbach α coefficient of the symptom items was 0.827.^[14]^

#### 2.4.5. Fatigue

The Chinese Patient‐Reported Outcome Measurement System–Breast–Chemotherapy fatigue short form was used to evaluate the level of cancer-related fatigue in participants. The fatigue short form consists of 12 items with a 5-point rating scale (1 = never or rarely, 5 = always). Participants were required to evaluate their fatigue experienced over the past 7 days. The total score ranges from 12 to 60, with higher scores indicating more severe levels of fatigue. The Chinese Patient‐Reported Outcome Measurement System–Breast–Chemotherapy was developed by Wu.^[15]^ The Cronbach α coefficient of the fatigue short form was 0.910.^[15]^

#### 2.4.6. Exercise self-efficacy

The Exercise Self-Efficacy Scale-Chinese version was used to assess the exercise self-efficacy of the participants. The scale consists of 9 items, and each item is rated on an 11-point Likert scale (0 = not very confident, 10 = very confident). The higher the total score, the higher the participants’ confidence in completing the exercise, that is, the higher the exercise self-efficacy. Application research on the Exercise Self-Efficacy Scale-Chinese version in Chinese BC patients has demonstrated that the scale has good reliability, with a Cronbach α coefficient of 0.86.^[16]^

#### 2.4.7. Depression

The Chinese version of the Patient-Reported Outcome Measurement Information System (PROMIS) depression short form was employed to assess depressive symptoms among participants. The depression short form consists of 8 items, each rated on a 5-point Likert scale. Participants were required to give responses based on their experiences with depression over a 7-day period. Higher scores indicate greater levels of depressive symptoms. The Cronbach α coefficient of the Chinese PROMIS depression short form was 0.88.^[17]^

#### 2.4.8. Social support

The Chinese PROMIS–social support short form was employed to measure the level of social support in participants. The social support short form was composed of 12 items covering 3 dimensions, namely instrumental support, emotional support, and informational support. Each item uses a 5-point self-report response format (1 = never or rarely, 5 = always). Total scores for the scale ranged from 12 to 60, with higher scores indicating better social support. The Cronbach α coefficients for all dimensions were >0.90.^[17]^

#### 2.4.9. Neighborhood built environment

The Chinese Walkable Environment Scale for urban community residents was employed to assess the neighborhood built environment in participants. The scale was composed of 17 items covering 5 dimensions, namely convenience of supporting living facilities, road conditions, neighborhood aesthetic, traffic conditions, and security. Convenience of supporting living facilities (4 items): evaluates whether essential facilities such as shops, public transportation stations, and parks are within walking distance. Road conditions (5 items): Assesses the road greening, cleanliness, smoothness, lighting, and walking exercise places. Neighborhood aesthetic (2 items): evaluates the aesthetic degree of the surrounding natural landscape and buildings. Traffic conditions (3 items): assesses traffic accidents, vehicle speeds, and sidewalk obstacles. Security (3 items): assesses perceived walking safety during the day and at night. Each item is rated on a 5-point Likert scale (1 = strongly agree, 5 = strongly disagree). The higher the total score, the better the perceived community walking environment. The Cronbach α coefficient of the scale was 0.807.^[18]^

### 2.5. Ethical considerations

The study conformed with the principles outlined in the Declaration of Helsinki and received approval from the Ethics Committee of the Second People’s Hospital of Changzhou (2024-KY027-01). All participants were presented with comprehensive information about the study before giving their consent. They were free to drop out of the study at any point without any negative effect on their care.

### 2.6. Statistical analysis

The data analyses were conducted using IBM SPSS Statistics Version 24.0 (IBM, Armonk). Sociodemographic and clinical characteristics are shown as frequencies with percentages for categorical variables. The measurement variables were examined for normality via histograms, as well as kurtosis and skew statistics, and reported as means with standard deviations (or median and interquartile range).

In univariate analysis, differences in SB between different sociodemographic and clinical groups were assessed by one-way analysis of variance, the Kruskal–Wallis *H* test, or independent-samples *t* test.

Pearson correlation analysis was used to analyze bivariate correlations between shoulder function, common cancer-related symptoms, fatigue, exercise self-efficacy, depression, social support, neighborhood built environment, and SB level, because these variables were normally distributed.

Then, candidate variables with a *P* value of <.05 were entered into the stepwise multiple linear regression model, with SB as the dependent variable. Specifically, categorical variables were quantified by dummy variables, and measurement variables were entered with actual scores. The significance level for all statistical tests was set at 0.05 (2-tailed).

## 3. Results

### 3.1. Characteristics of participants

A total of 234 recruited BC patients were eligible, and all of them completed the survey. The average age of participants was 53.74 ± 11.25 years. Educational level distribution was as follows: 52 (22.22%) participants had primary school education or below, 85 (36.32%) participants completed middle school, 43 (18.38%) participants finished high school, and 54 (23.08%) participants held college degrees or above. The majority of participants (83.76%) were married, and 214 (91.45%) lived with other residents. Two hundred and ten (89.74%) participants were unemployed or retired. Almost half of participants (48.72%) were overweight. Ninety-seven (41.45%) participants were diagnosed with stage II cancer, 138 (58.97%) participants underwent mastectomy, and 153 (65.38%) participants had no comorbidities. The chemotherapy courses completed by the participants ranged from 1 to 8 courses (Table 1).

**Table 1:** Sedentary behavior of different participant characteristics (N = 234).

Variable	N (%)	Sedentary behavior		
		Mean + SD	Test value	*P* value
Age (yr)			3.907*	.021
18–44	52 (22.22)	6.41 ± 1.20		
45–59	109 (46.58)	5.83 ± 1.30		
≥60	73 (31.20)	6.19 ± 1.41		
Educational level			6.423†	.093
Primary school or below	52 (22.22)	6.03 ± 1.23		
Middle school	85 (36.32)	6.04 ± 1.47		
High school	43 (18.38)	5.81 ± 1.20		
College or above	54 (23.08)	6.36 ± 1.29		
Marital status			11.822*	<.001
Married	196 (83.76)	5.90 ± 1.36		
Single	7 (2.99)	7.36 ± 0.88		
Divorced or widowed	31 (13.25)	6.89 ± 0.60		
Residence status			−6.918‡	<.001
Living with other residents	214 (91.45)	5.98 ± 1.34		
Living alone	20 (8.55)	7.11 ± 0.61		
Work status			−2.410‡	.017
Unemployed or retired	210 (89.74)	6.00 ± 1.35		
Employed	24 (10.26)	6.69 ± 0.99		
Average monthly household income (yuan, RMB)			1.513*	.212
<3000	79 (33.76)	6.21 ± 1.21		
3000–5999	86 (36.75)	6.11 ± 1.27		
6000–8999	59 (25.22)	5.77 ± 1.60		
≥9000	10 (4.27)	6.39 ± 0.76		
Body mass index (kg/m^2^)			7.482†	.024
<18.5	5 (2.14)	5.19 ± 0.56		
18.5–23.9	115 (49.14)	5.95 ± 1.36		
≥24.0	114 (48.72)	6.23 ± 1.31		
The number of comorbidities			0.561†	.755
0	153 (65.38)	6.01 ± 1.39		
1	58 (24.79)	6.13 ± 1.26		
2 or above	23 (9.83)	6.31 ± 1.08		
History of surgery			1.550*	.215
Non	45 (19.23)	5.77 ± 1.59		
Lumpectomy	51 (21.80)	6.08 ± 1.22		
Mastectomy	138 (58.97)	6.17 ± 1.27		
Cancer stage			4.680*	.010
I	88 (37.61)	6.15 ± 1.26		
II	97 (41.45)	6.26 ± 1.18		
III	49 (20.94)	5.57 ± 1.62		
The number of completed chemotherapy courses			1.419*	.238
1–2	103 (44.02)	6.25 ± 1.23		
3–4	73 (31.19)	6.02 ± 1.18		
5–6	39 (16.67)	5.92 ± 1.59		
≥7	19 (8.12)	5.66 ± 1.77		

ANOVA = analysis of variance.

* One-way ANOVA test.

† Kruskal–Wallis *H* test.

‡ Independent-samples *t* test.

### 3.2. Difference in SB among participants with different characteristics

Univariate analysis identified sociodemographic characteristics (age, current marital status, residence status, work status) and clinical characteristics (BMI, cancer stage) as potential determinants of SB (Table 1).

### 3.3. Observed variables scores and the bivariate correlations with SB

The average daily sedentary time of BC patients during chemotherapy was 6.07 ± 1.33 hours (range: 1.50–8.50). The average scores for the remaining variables were as follows: exercise self-efficacy, 31.23 ± 6.80; depression, 13.15 ± 4.08; common cancer-related symptoms, 30.27 ± 6.60; fatigue, 16.18 ± 3.40; shoulder function, 88.83 ± 8.70; social support, 45.18 ± 5.94; neighborhood built environment, 74.08 ± 3.37. The average scores of the 3 dimensions of social support, namely instrumental support, informational support, and emotional support, were 15.19 ± 2.42, 14.80 ± 2.52, and 15.20 ± 2.33, respectively (Table 2).

**Table 2:** Association of other measurement indexes and sedentary behavior (N = 234).

Variables	Score	Correlation coefficient	*P* value
Exercise self-efficacy	31.23 ± 6.80	−0.200	.002
Depression	13.15 ± 4.08	0.234	<.001
Common cancer-related symptoms	30.27 ± 6.60	0.153	.019
Fatigue	16.18 ± 3.40	0.193	.003
Shoulder function	88.83 ± 8.70	−0.175	.007
Social support	45.18 ± 5.94	−0.006	.928
Instrumental support	15.19 ± 2.42	0.201	.002
Information support	14.80 ± 2.52	−0.131	.045
Emotional support	15.20 ± 2.33	−0.082	.210
Neighborhood built environment	74.08 ± 3.37	−0.058	.381

Correlation analysis showed that SB was positively correlated with depression (*r* = 0.234, *P* < .001), common cancer-related symptoms (*r* = 0.153, *P* = .019), and fatigue (*r* = 0.193, *P* = .003), but negatively correlated with exercise self-efficacy (*r* = −0.200, *P* = .002) and shoulder function (*r* = −0.175, *P* = .007). Unexpectedly, no significant correlations were observed between SB and social support or the neighborhood built environment (*P* > .05). Further investigation into different dimensions of social support revealed that only instrumental support (*r* = 0.201, *P* = .002) and informational support (*r* = −0.131, *P* = .045) had a significant correlation with SB (Table 2).

### 3.4. Determinants of SB

Age, residence status, work status, BMI, cancer stage, exercise self-efficacy, depression, fatigue, shoulder function, instrumental support, and informational support (all *P* < .05) were entered into the stepwise multiple linear regression model. As shown in Table 3, these variables collectively explained 33.46% of the variance in SB.

**Table 3:** Linear regression analysis of factors influencing sedentary behavior in breast cancer patients during chemotherapy (N = 234).

Variables	*B*	SE	*β*	*t*	*P* value	*R* ^2^	*F*
(Constant)	4.796	1.489		3.221	.001	33.46%	7.893
Age (yr) (compared to 18–44)							
45–59	−0.626	0.222	−0.235	−2.825	.005		
≥60	−0.539	0.273	−0.188	−1.975	.049		
Marital status (compared to married)							
Single	0.349	0.485	0.045	0.720	.472		
Divorced or widowed	0.156	0.315	0.040	0.494	.621		
Residence status (compared to living with other residents)							
Living alone	1.062	0.395	0.224	2.688	.008		
Work status (compared to Unemployed or retired)							
Employed	0.831	0.260	0.190	3.200	.002		
Body mass index (compared to <18.5 kg/m^2^)							
18.5–23.9 kg/m^2^	1.104	0.517	0.415	2.135	.034		
≥24.0 kg/m^2^	1.312	0.512	0.494	2.562	.011		
Cancer stage (compared to I)							
II	0.069	0.171	0.026	0.403	.687		
III	−0.465	0.204	−0.143	−2.282	.023		
Exercise self-efficacy (score)	−0.025	0.012	−0.125	−2.024	.044		
Depression (score)	0.053	0.022	0.162	2.434	.016		
Common cancer-related symptoms (score)	0.011	0.013	0.054	0.843	.400		
Fatigue (score)	0.058	0.024	0.148	2.435	.016		
Shoulder function (score)	−0.020	0.010	−0.130	−1.985	.048		
Instrumental support (score)	0.156	0.036	0.284	4.385	<.001		
Information support (score)	−0.093	0.042	−0.176	−2.239	.026		

Variables entered with stepwise entry.

*β* = standardized beta coefficients, *B* = unstandardized beta coefficients, *R*^2^ = adjusted *R*^2^, SE = standard error.

Participants aged 45 to 59 years (*β* = −0.235, *P* = .005) and ≥60 years (*β* = −0.188, *P* = .049) tended to have less SB than participants aged 18 to 44 years. Participants who lived alone (*β* = 0.224, *P* = .008) and those who were employed (*β* = 0.190, *P* = .002) tended to increase SB. Compared with BMI < 18.5 kg/m^2^, participants with a BMI of 18.5 to 23.9 kg/m^2^ (*β* = 0.415, *P* = .034) and ≥24.0 kg/m^2^ (*β* = 0.494, *P* = .011) tended to have higher levels of SB. Compared with cancer stage I, cancer stage III was often associated with lower levels of SB (*β* = −0.143, *P* = .023). In addition, higher depression, fatigue, and instrumental support scores were often associated with increased SB. Conversely, higher exercise self-efficacy, shoulder function, and informational support scores were associated with decreased SB (Table 3).

## 4. Discussion

Although SB has been identified as an important behavioral intervention target in recent years, this survey shows that SB is prevalent in BC patients during chemotherapy, with participants self-reporting an average of 6.07 ± 1.33 hours of daily sedentary time. The present results align closely with the observations made by Khandalavala et al in their American cohort.^[19]^ Considering that major treatment phases, such as chemotherapy, may be pivotal periods for modifications in the health behaviors of cancer patients, it is particularly important to understand the influencing factors of SB during chemotherapy. Based on the COM-B model and the variable mechanisms identified through a literature review, this study attempts to clarify the key determinants of SB in BC patients during chemotherapy from 3 aspects: capability, opportunity, and motivation. The findings indicate that age, residential status, work status, BMI, cancer stage, depression, fatigue, exercise self-efficacy, shoulder function, instrumental support, and informational support were independent predictors of self-reported SB. These findings can be used to guide future interventions to reduce SB in BC patients during chemotherapy.

### 4.1. Motivation and SB

For the motivation domain, our findings indicate that exercise self-efficacy is an important factor influencing the SB of BC patients during chemotherapy. This discovery is theoretically supported by the COM-B model and the self-determination theory, which reveal that behaviors are primarily driven by individuals’ motivation.^[20]^ If individuals have confidence in their ability to perform a particular behavior (self-efficacy), they will be driven by that confidence, more inclined to participate in such behavior and persist despite challenges.^[21]^ Research results from different populations have confirmed that higher levels of self-efficacy can help stimulate automated exercise behaviors and promote long-term adherence while shortening sedentary time.^[6,22,23]^ In this study, the exercise self-efficacy of the patients was at a relatively low level. This may be influenced by the traditional Chinese belief that the body remains in a weak state during chemotherapy, which may weaken their confidence in participating in physical activities. Evidence from Japanese populations indicates that self-efficacy served as a significant determinant of physical activity engagement, while over 60% of BC survivors reported leisure-time inactivity.^[24]^ Therefore, nurses should pay attention to the assessment of exercise self-efficacy. For patients with low self-efficacy levels, appropriate intervention strategies can be formulated, such as explaining the significance of increasing physical activity and associated knowledge, organizing group rehabilitation exercises in the ward environment to help patients accumulate physical activity experience, and provide positive feedback on exercise behaviors. These measures aim to enhance their confidence in physical activities, thereby reducing SB.

This study also found that the level of depression was positively correlated with the daily sedentary time of BC patients during chemotherapy, which was supported by a previous study.^[25]^ A possible explanation is that patients with depression have persistent low moods, lose interest in pleasant activities, and generally decline in energy levels, resulting in reduced social participation and a greater tendency towards a sedentary lifestyle. Additionally, studies have indicated that prolonged SB is also an independent risk factor for depression, thereby forming a vicious cycle of depression-SB-depression.^[26]^ Therefore, for patients with depressive tendencies, nurses should encourage them to actively participate in social activities suitable for them, stimulate their interest in physical activities, reduce sedentary time while also alleviating depression.

### 4.2. Capability and SB

According to the COM-B model, capability is a prerequisite for persisting in physical activity and for reducing SB.^[22]^ For the capability domain, our findings showed that declined shoulder function in the affected limb and increased fatigue levels were key factors promoting SB in patients. Impaired shoulder function on the affected side is the earliest and most common postoperative complication in BC patients, commonly presenting as joint pain and reduced range of motion.^[27,28]^ Since the shoulder joint bears the primary load for most upper extremity motions, when its function declines, patients’ daily activities such as dressing, cooking, cleaning, and participating in sports are inevitably restricted.^[28]^ Some patients consciously limit their activities to ensure safety and avoid exacerbating shoulder function injuries due to inappropriate activities.^[29]^ These findings suggest that we should pay attention to the recovery of shoulder function in the affected limb, develop a scientific and standardized shoulder rehabilitation plan in combination with physical therapists, and enhance patients’ positive health beliefs.

A previous study indicated that the duration of fatigue was positively correlated with SB among BC survivors, consistent with the results of this study.^[30]^ Fatigue is one of the most common and distressing complaints reported by cancer patients during chemotherapy, and it may persist even after the end of chemotherapy.^[31]^ In spite of this, some BC patients still have cognitive misunderstandings about fatigue management and attempt to relieve fatigue symptoms through prolonged SB. On the contrary, the longer the sedentary time, the higher the degree of subsequent fatigue.^[25]^ Therefore, nurses should pay close attention to the perception and self-management of fatigue in BC patients, and formulate individualized intervention strategies tailored to this population.

### 4.3. Opportunity and SB

According to the COM-B model, opportunities represent external factors that promote behavior.^[22]^ For the opportunity domain, this study examined the influence of neighborhood built environment and social support on SB. The results showed that there was no significant statistical association between either of them and sedentary time. Further, we specifically examined the influences of the 3 dimensions of social support (instrumental support, emotional support, and informational support) on SB respectively. The results demonstrated contrasting effects of informational and instrumental support on SB: effective informational support contributed to a reduction in SB, whereas greater instrumental support was associated with an increase in SB. Meanwhile, emotional support had no significant association with sedentary time. This may be because effective informational support helps enhance patients’ access to health behavior-related knowledge, helps them better understand the negative impacts of SB and how to replace it with physical activity, thereby promoting patients’ awareness of modifying sedentary habits. On the contrary, patients with high levels of instrumental support enjoy more alternative services provided by others in terms of daily life, household chores, and financial assistance, etc. This overprotection, to some extent, hinders their physical activity participation and increases their tendency to remain sedentary for extended periods. Previous studies have shown that positive social relations and emotional support can help BC survivors effectively cope with stress and alleviate their negative emotions.^[32]^ Based on this, some scholars speculate that emotional support may reduce SB by regulating negative emotions.^[33]^ However, this point has not been directly verified in this study, possibly because the expression of emotional support in Chinese culture is often accompanied by reminders to rest.

In addition, studies on older adults have shown a significant negative correlation between SB patterns and the neighborhood environment.^[34]^ In this study, the relationship was not statistically significant. It may be due to the fact that all participants originated from an economically developed city in southeastern China, where the community environment was generally favorable. Consequently, the distribution of scores related to this measure was concentrated and generally high, potentially leading to a ceiling effect and reduced statistical sensitivity. Future research should consider multicenter studies to further examine the association between SB and the community environment.

### 4.4. Other factors associated with SB

This study found that middle-aged and elderly BC patients during chemotherapy had shorter sedentary time compared to young patients. This finding aligns with a previous study indicating that young individuals had a higher incidence of engaging in SB for more than 6 hours daily.^[35]^ Notably, some studies had yielded conflicting findings that older individuals tend to have higher levels of sedentariness.^[36]^ Overall, there was no consistent evidence to support associations between SB and age. Additionally, postmenopausal women may pay more attention to healthy lifestyles and consider exercise an important component of their routine.^[37]^ This phenomenon partially accounted for our finding. Sociological factors, including living alone and remaining employed, were also identified as determinants of SB. This finding was supported by research conducted by Gavin et al.^[6]^ Patients living alone exhibit a high level of SB, which may be due to their lack of peer support and limited, static social networks, resulting in less physical activity. Patients who remain employed not only have sedentary leisure time but also tend to be sedentary at work. Consistent with previous studies, this study also showed that increased BMI is a risk factor for SB.^[6]^ Furthermore, previous studies have shown that individuals with advanced-stage BC are prone to long-term SB.^[5]^ However, this study revealed that BC patients with stage III tumors exhibited significantly reduced sedentary time compared to those with stage I tumors. This may be because patients with stage III tumors perceive greater health risks, which drives them to actively reduce unhealthy behaviors such as SB. In summary, these findings provide additional references for nurses to identify high-risk groups for SB. Nurses should pay particular attention to BC patients undergoing chemotherapy who are young, remain employed, live alone, have a high BMI, or have stage I tumors. They should formulate people-oriented intervention plans early, provide knowledge related to SB, and prompt them to understand the possible harms caused by SB.

### 4.5. Limitations

This study has several limitations. Firstly, sedentary time was collected through self-reporting methods, which may introduce expectation bias and recall bias, thereby compromising the reliability of the results. Secondly, the participants in this study were recruited from a large general hospital in China, which may result in insufficient sample representativeness. Therefore, generalizability of our findings to more diverse populations might be limited. Finally, the cross-sectional design used in this study limits the ability to infer causality. Therefore, further longitudinal and experimental studies are needed in the future to substantiate and expand upon the findings presented herein.

## 5. Conclusions

SB is an important health-related behavior problem that is prevalent among BC patients during chemotherapy. The use of the COM-B model as an organizational framework helps to identify determinants of physical inactivity in this population. The results showed that exercise self-efficacy, depression, fatigue, shoulder function, instrumental support, informational support, age, residence status, work status, BMI, and cancer stage were independent influencing factors of SB. As a practical implication of our results, future interventions seeking to reduce SB in BC patients during chemotherapy could be designed and evaluated based on the above identified factors.

## Acknowledgments

The authors would like to give their gratitude to the volunteers who participated in the study.

## Author contributions

**Conceptualization:** Ye Yang, Wei Jia.

**Data curation:** Wei Jia.

**Formal analysis:** Wei Jia, Lingyun Xu.

**Investigation:** Ye Yang, Kaiping Wu.

**Methodology:** Ye Yang, Wei Jia, Yuwen Jiao.

**Project administration:** Ye Yang, Kaiping Wu.

**Supervision:** Kaiping Wu.

**Visualization:** Wei Jia, Lingyun Xu.

**Writing – original draft:** Ye Yang, Wei Jia, Yuwen Jiao.

**Writing – review & editing:** Ye Yang, Wei Jia, Lingyun Xu.
